# Supplementary figures and images for: Competition among variants is predictable and contributes to the antigenic variation dynamics of African trypanosomes
Source: PLoS Pathog. 2023 Jul 17;19(7):e1011530. doi: 10.1371/journal.ppat.1011530 (PMC10374056; doi:10.1371/journal.ppat.1011530)

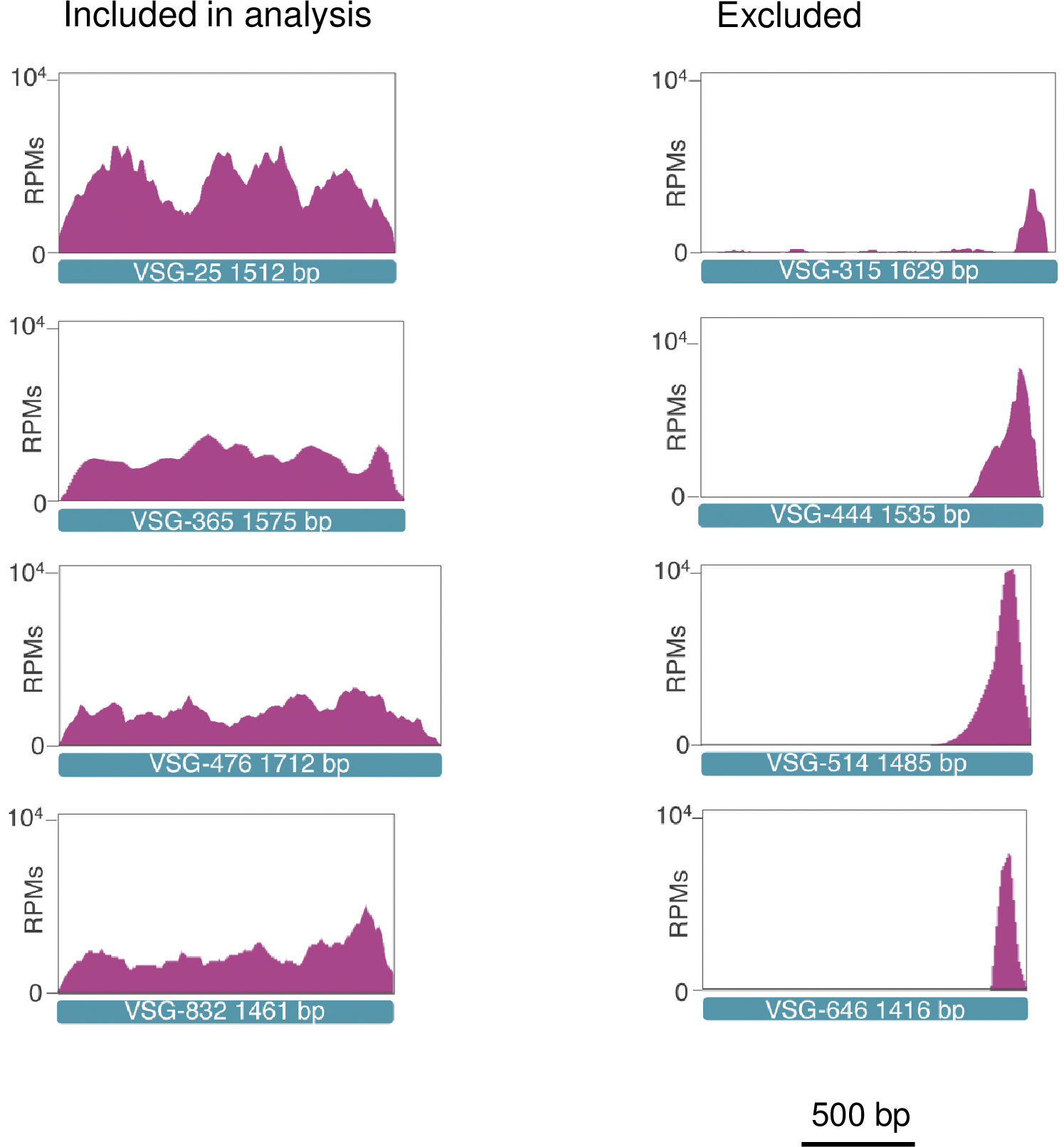

Supplement: S1 Fig — The VSG set was filtered based on RNA-seq read-mapping profiles. VSGs displaying full coding sequence activation were included in the analysis. VSGs with mapped reads restricted to the C-terminal coding sequence were excluded from further analysis. These results are explained by the presence of common sequences in multiple VSGs. The 3’-terminal 268 nucleotides of VSG-444 are shared with VSG-18, while the 3’-terminal 584 nucleotides of VSG-514 are shared with VSG-17, for example. VSGs included in the analysis were truncated to 1,200 bp to remove shared sequence. (TIF) [file ppat.1011530.s001.tif]

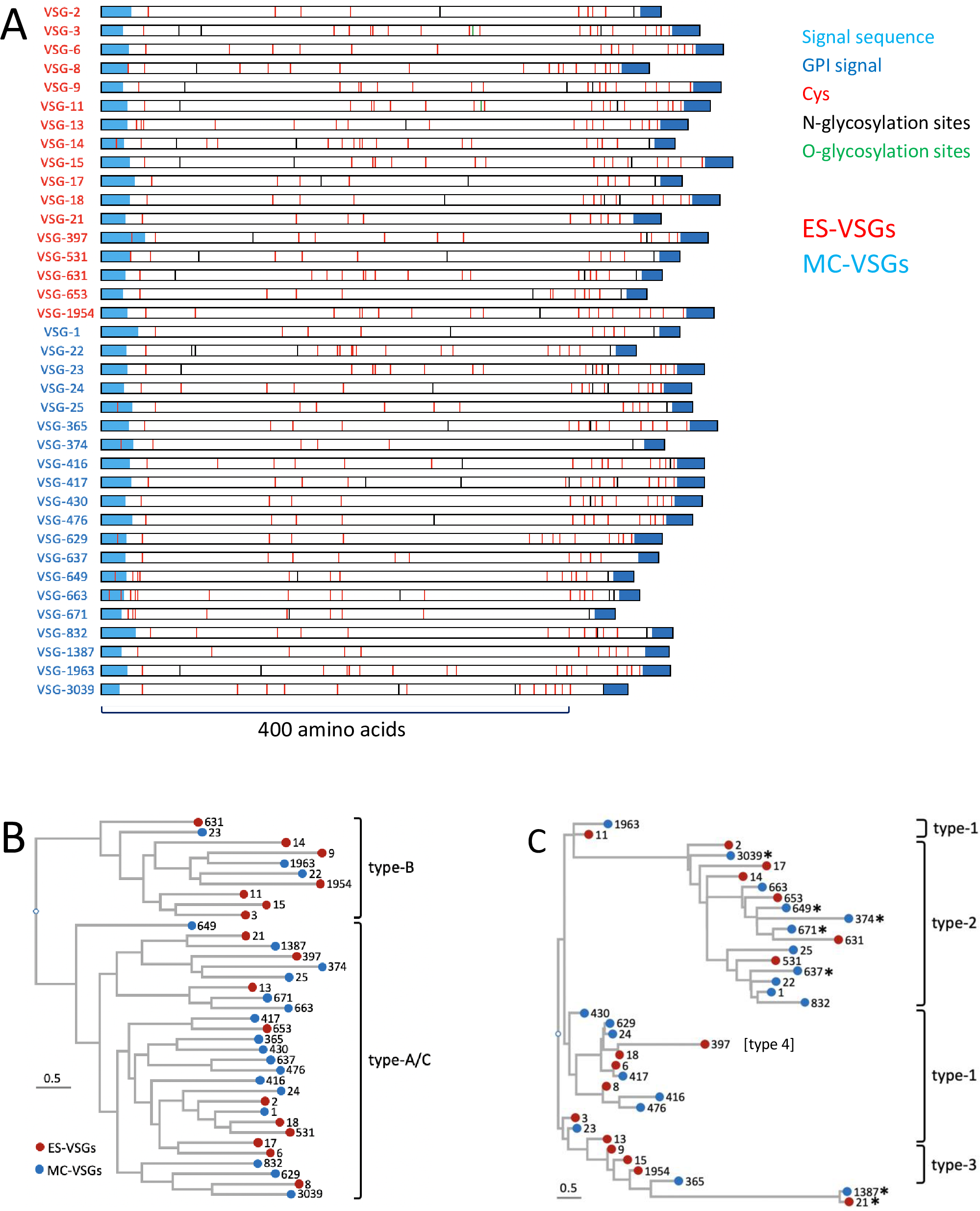

Supplement: S2 Fig — (A) The schematic shows the activated set of VSG proteins with salient features highlighted. Signal peptides were predicted using the SignalP tool. GPI modification sites were predicted using the big-PI predictor. N-glycosylation sites were predicted using NetNGlyc. O-glycosylation sites are from [32]. VSG domain types are indicated. (B) The phylogenetic tree shows the activated set of VSGs analyzed using ETE 3 [53]; the N-terminal 400 amino acids only. (C) As in B but for the C-terminal 100 amino acids only. An asterisk indicates undefined VSG C-terminal type. (TIF) [file ppat.1011530.s002.tif]

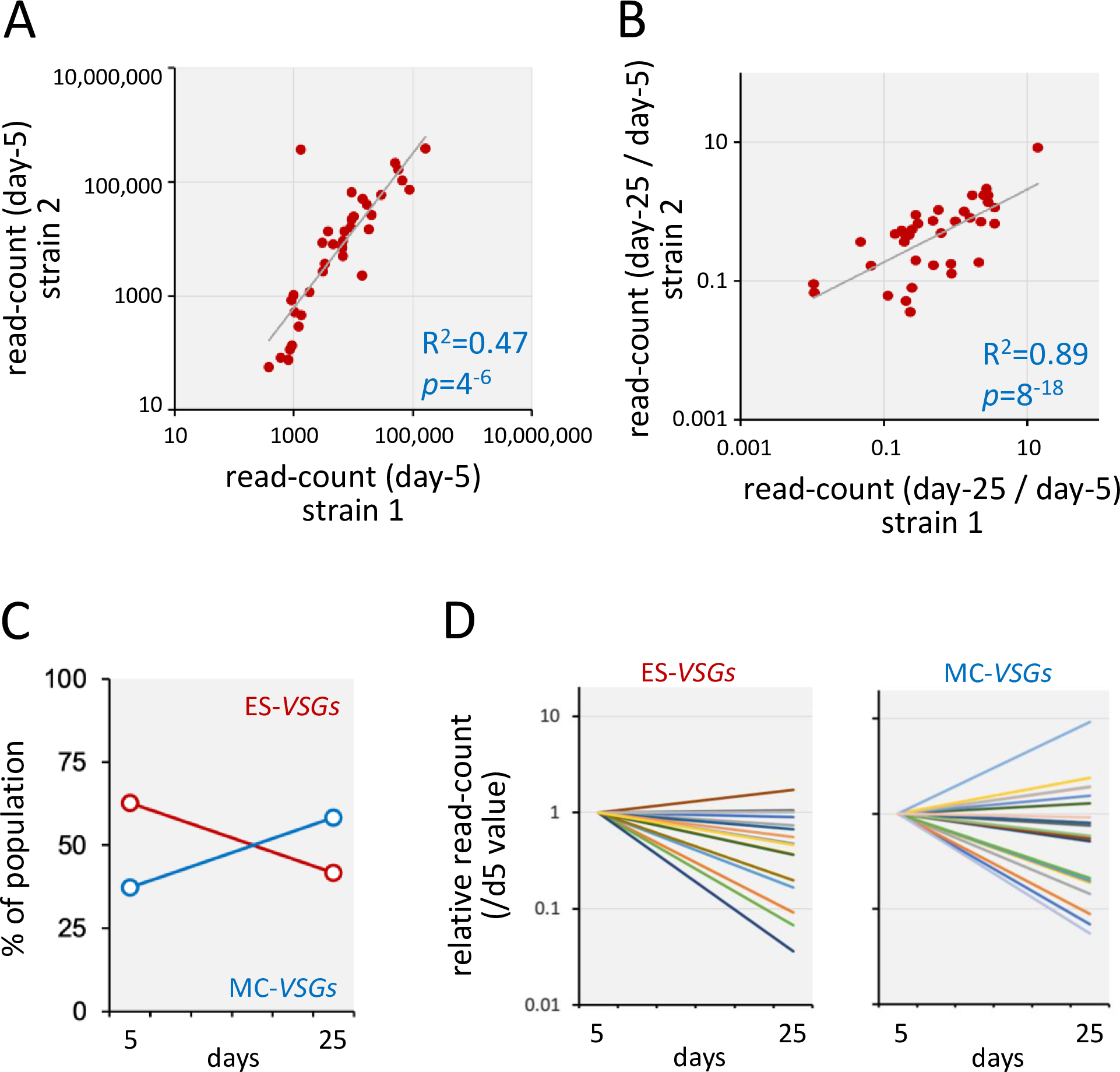

Supplement: S3 Fig — (A) The graph shows VSG expression levels as determined by RNA-seq read-count on day 5 after inducing switching in two independent biological replicate strains. n = 36 VSGs. (B) The graph shows relative VSG expression levels as determined by relative RNA-seq read-count over 20 days of growth following switching in two independent biological replicate strains. n = 36 VSGs. (C) Relative read-counts for ES-VSGs (n = 16) and MC-VSGs (n = 20) at day-5 and day-25 in the second replicate strain, and as determined by RNA-seq; three replicates, error bars, SD (not visible). (D) Read-counts for individual ES-VSGs (n = 16) and MC-VSGs (n = 20) at day-25 relative to the day-5 values in the second replicate strain. (TIF) [file ppat.1011530.s003.tif]

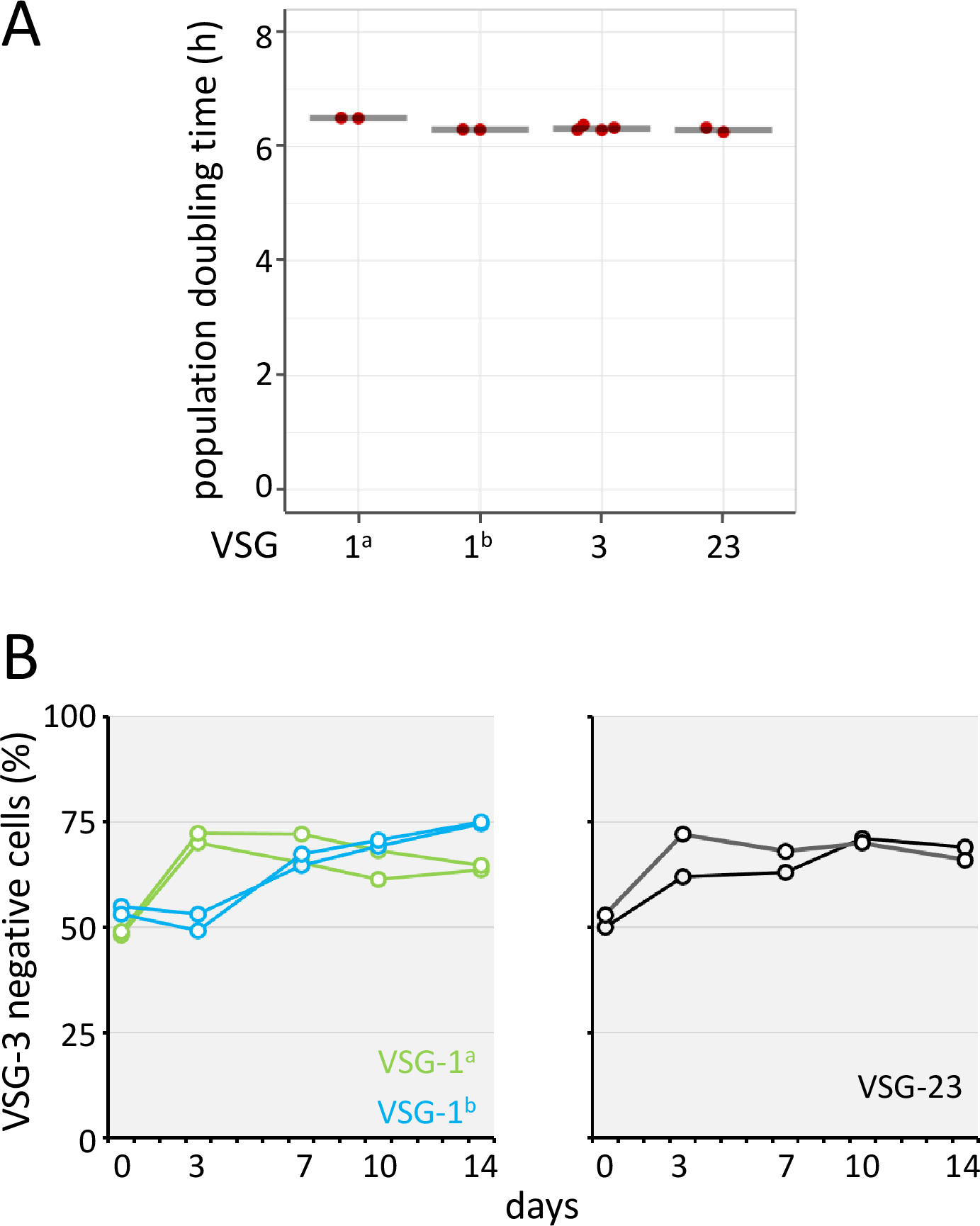

Supplement: S4 Fig — (A) The graph shows doubling times calculated for individual clones expressing the VSGs indicated. Duplicate flasks were counted after three days of growth; quadruplicate flasks in the case of the VSG-3 expressing clone. The horizontal bars indicate average values. (B) The VSG-3 expressing cells were mixed 50:50 with each of the other clones shown in A. Cells were then stained with αVSG-3 on the days indicated, and the proportion of VSG-3 negative cells were counted by microscopy. (TIF) [file ppat.1011530.s004.tif]
