# Supplementary material for: Competition among variants is predictable and contributes to the antigenic variation dynamics of African trypanosomes
Source: PLoS Pathog. 2023 Jul 17;19(7):e1011530. doi: 10.1371/journal.ppat.1011530 (PMC10374056; doi:10.1371/journal.ppat.1011530)
Supplement: Attachment — Submitted filename: Escrivani Scheidt responses.pdf [file ppat.1011530.s006.pdf]

PLOS Pathogens
Competition among variants is predictable and contributes to the antigenic variation dynamics of African trypanosomes

Dear Nina,
Thank-you for the feedback on our manuscript and apologies for the delay in submitting the revised version. We also thank all of the reviewers for their comments. As suggested by reviewer 1, we generated a new strain for further RNA-seq analysis and report new data using this independent biological replicate, showing that our major findings are reproducible; 'that antigenic variants compete, and that the population of cells that activates minichromosome derived *VSGs* displays a competitive advantage' (Abstract). We have responded to all comments below, and new data have also now been included to address some of reviewer 2's comments. We hope that our paper is now acceptable for publication.
Best wishes,
David

Part I – Summary

Reviewer #1:
1.1: In this paper, Scheidt and Escrivani et al demonstrate that T brucei populations composed of parasites expressing multiple VSGs show predictable patterns of VSG expression in vitro over time, in the absence of a host immune response. They find that initially expression-site associated VSGs emerge, but later minichromosomal VSGs establish within the populations. There is a slight correlation between parasite fitness/outgrowth and VSG length, and slow-growing parasite clones show transcriptional differences compared to faster ones. Overall, their data support the idea that competition between different VSG-expressing subpopulations plays a role in this parasite's ability to evade host adaptive immunity for very long periods of time, which is novel and interesting. The paper would be greatly improved by the use of biological replicates (multiple parasite clones and multiple break inductions) to support their findings.
R1.1: We were happy to see that reviewer 1 finds our results 'novel and interesting'. We also agree with the comment in relation to 'biological replicates'. To address this point, we 'generated another independent 'sgRNA break-site 3' strain, induced VSG switching, and carried out RNA-seq analysis on days-5 and -25. The results, described in the 'Switched populations…' Results section, and shown in Supplementary Fig S3, confirm both major findings, 'that antigenic variants compete, and that the population of cells that activates minichromosome derived *VSGs* displays a competitive advantage' (see the Abstract).

Reviewer #2:
2.1: In this work, the authors use CRISPR technology and genome-wide RNA sequencing to tackle an old question in the antigenic variation field: whether post-VSG switching populations compete with each other, thus contributing to the global predicted pattern of VSG expression during infection. The authors characterized the dynamics of the heterogenous VSG-switched population over time (day 5 to 25 post-VSG switch). Parasites expressing VSG from Mini-chromosomes became predominant in the switched population, while VSG from Expression Sites (ES) became rarer. This effect was associated with the fact that the ES-VSG were encoded by a longer gene. More careful analysis of the full transcriptome revealed that parasites that activate long ES-VSG undergo a transient reduction in growth (day 9), which is no longer detectable at day 25. The authors do not study how expression of a longer VSG could affect growth rate, but they propose that such reduced transient growth could be beneficial for not triggering a strong host immune system immediately after VSG switching.

This works has an original angle on antigenic variation, because it focuses on the population dynamics after switching instead of studying the switching moment or steady-state conditions

where VSGs are maintained silent/active. The paper is very well written and the figures are extremely clear.
R2.1: We thank this reviewer for their positive comments.

Reviewer #3:
3.1: This manuscript by Scheidt and colleagues provides an analysis of the parameters that affect activation and/or abundance of Variant Surface Glycoprotein (VSG) during antigenic variation by Trypanosoma brucei. The approach taken is to model a switch in expressed VSG by conditional expression of Cas9 endonuclease, targeting a DNA double strand break around the transcribed VSG gene within the bloodstream VSG expression site. While the parameters affecting the efficiency of VSG selection/expression are thoroughly and clearly explored, I'm afraid that I find the extensive discussion and conclusions reached by the authors overreach the constraints of the assay system adopted.
The two major conclusions reached are that VSG length dictates VSG activation efficiency or abundance (it is unclear which they favour), and that differential growth rates of parasites newly expressing distinct VSGs is reflected in transcriptome differences. As explained below, both conclusions are insecurely grounded in the available data. In addition, in the discussion, the authors make further claims from their experiments that have not been tested adequately.
R3.1: We thank this reviewer for their comments. In our view, however, our two major findings/conclusions are, 'that antigenic variants compete, and that the population of cells that activates minichromosome derived *VSGs* displays a competitive advantage'. Both findings have now been confirmed using an independent biological replicate (see above). In relation to VSG length, see our Results section, 'VSG dynamics in switched populations transiently correlate with VSG length'; where we state 'VSG activation frequency (Fig 4D) … failed to achieve a significant correlation with VSG length for either cohort of VSGs'. Indeed, we also state in the Discussion that 'Our findings, however, do not provide support for VSG length-dependent activation rates'. We address further comments in relation to 'transcriptome differences' below (see R3.6).

Part II – Major Issues: Key Experiments Required for Acceptance

Reviewer #1:
1.2: Were all the experiments performed using one sgRNA clone? From the text this appears to be the case. If so, one cannot rule out (however unlikely) that the patterns observed are clone-specific. it would be good to know that these patterns transcend the individual clone. At the very least it would be important to acknowledge the possibility that individual clones may have different intrinsic tendencies.
R1.2: Yes, and we agree that 'it would be good to know that these patterns transcend the individual clone'. We, therefore, 'generated another independent 'sgRNA break-site 3' strain, induced VSG switching, and carried out RNA-seq analysis on days-5 and -25'. The results confirm our major findings (see R1.1 above, Fig S3 and the new text in the 'Switched populations…' Results section).

1.3: - Similarly, using technical replicates for the RNA-seq, instead of three separate break inductions, might reveal an induction-specific set of outcomes that wouldn't occur every time a cut is made, even though the three parallel cultures follow the same trajectory. The fact that the second induction of a break (data in figure 5C) resulted in a slightly different set of clones, further raises suspicion that this pattern might be more variable than the initial experiment suggests. The authors state that this result reflects under-sampling, possibly due to growth differences influencing clonal expansion, but without parallel RNA-seq data it is hard to be sure this is truly under sampling and not a slightly different experimental outcome.
R1.3: As detailed above, we repeated the RNA-seq analysis with an independent clone and show that patterns of VSG activation and subsequent dynamics are remarkably reproducible (see R1.2 above).

Reviewer #2:
2.2: Most conclusions are well supported by the findings. I have three questions related to Figure 5, from which authors conclude that growth rate appears to be transiently reduced when parasites switch from VSG-2 to a longer VSG (ES-VSGs).
1. On day 25, the authors no longer detect the temporary reduction of transcript levels related to energy metabolism and translation first detected in day 9, suggesting that on day 25 all switchers proliferate at similar rates. This result should be directly demonstrated by either measuring growth rate of individual subclones isolated on day 25 (expressing different VSGs, of different chromosomal locations and lengths) or, even better, by doing competition experiments between such subclones.
R2.2: To clarify here, the transcriptome changes shown in Fig 5B may be explained by reduced relative abundance of slower-growing cells. Nevertheless, we measured growth rates for individual subclones and ran some competition experiments. The new results are described in the 'Switched populations…' Results section and are shown in Supplementary Fig S4. The observations are notably consistent with reports on competition among variants described over forty years ago.

2.3: 2. It is not clear how growth rate was measured from the RNAseq data. Typically the units for cell proliferation are in time/division. While the evidence of a temporary reduction of transcript levels related to energy metabolism and translation is compelling and consistent with a transient reduced growth rate, the authors' conclusion on transient reduced growth rate would be more robust if they could directly measure proliferation rate of individual switchers over time. It seems this could be achieved by flow cytometry analysis of parasite population post-switching using VSG antibodies (VSG-9, VSG-6, VSG-13, VSG-3) and Proliferation dies (such as Cell Trace Violet) at multiple days post-induction.
R2.3: We now provide some measures of growth rates for individual subclones and express these as time/division (see Supplementary Fig S4A).

2.4: 3. I wonder if the transient reduced growth rate is because parasites need to adjust from translating/expressing a small VSG (VSG-2) to a large VSG. Would the same transient reduced growth rate be observed if the starting parasite population expressed a large VSG (such as VSG-6 or VSG-8) instead of VSG-2, and switched to another large VSG? What would happen to proliferation if parasites switched from a large to a short VSG?
R2.4: We would need to repeat the analysis with a new 'Cas9-sgRNA' strain expressing a larger VSG to address these questions, not something we can deliver quickly unfortunately. Ultimately, we suspect that the pre-switch VSG has little to no impact on subsequent dynamics, and that cells expressing minichromosome derived VSGs would maintain a competitive advantage. Some of our thinking here is detailed in our Discussion, but we're afraid we cannot directly answer these questions at present.

Reviewer #3:
3.2: 1. The authors conclude that, amongst a range of factors that affect VSG abundance after induced switching (including genomic location and flanking sequence homology), is VSG coding sequence length. As stated above, the authors need to make clear what their RNA-seq approach provides a readout for: activation probability, or an effect of the expressed VSG on growth? For instance, in the discussion they state: 'RNA-seq analysis then provided a measure of relative VSG activation frequency and relative subsequent growth rate for cells expressing different VSGs'. They also state: 'Our findings, however, do not provide any support for VSG length-dependent activation rates'. Clearly these two processes are not equivalent and require differing hypotheses to explain the data. It seems the authors infer that RNA-seq at a single time point is a measure of activation, while comparison of two time points is a measure of relative growth, but it is not explained why these effects can be inferred in this way.

R3.2: We've attempted to clarify by editing this statement in the Discussion: "RNA-seq analysis then provided a measure of relative VSG activation frequency, at the earliest post-switching time-point, and relative growth rate, at subsequent time-points". Note that we also state in the second Results section, "An assessment of relative read-count in our earliest RNA-seq samples (day 5) then revealed differential activation frequencies for each *VSG* and for each cohort of *VSGs* (Fig 2D)". The statement, 'Our findings, however, do not provide support for VSG length-dependent activation rates', is included in relation to an observation from a prior mathematical modelling study, which 'indicated that VSG length-dependent activation rates have the potential to allow *T. brucei* to persist for moderately longer in vivo [17]'. We do indeed infer 'that RNA-seq at a single time point [provides] a measure of activation, while comparison of two time points [provides] a measure of relative growth' and we have now attempted to explain our reasoning more clearly. See the reference to the Materials and Methods section in the third paragraph of the 'RNA-seq revealed…' Results section, the new text towards the beginning of the 'Switched populations…' Results section, and the new text in the 'RNA sequencing…' Materials and Methods section. Silent VSGs typically produce little RNA (<0.002% of the total), such that a signal 10-fold above 'background' can be used to quantify new variants present in the population.

3.3: Irrespective, the data presented is contradictory for an influence of VSG length, and hence there seems to be no compelling case for such a simple relationship:
R3.3: Apologies, but we don't see a contradiction. The data presented in Fig 3D support the view that VSG length has a (statistically significant) impact on the competition among variants.

3.4: Fig.3D presents the main evidence for this conclusion but reveals an opposite correlation between abundance and length of VSGs found in silent expression sites (shorter = greater abundance) and in minichromosomes (longer = greater abundance). If VSG length is, as suggested, a primary determinant of VSG usage, the authors need to provide an explanation for why shorter VSGs in one location are activated/grow more efficiently, while longer VSGs are activated/grow more efficiently in another. The only explanation provided appears to make no sense: 'What is less clear is why cells expressing longer minichromosome-derived VSGs appear to grow faster'. All VSGs are expressed from the same location (the VSG expression site), so why should the silent location from which they are sourced have any influence on growth, rather than activation efficiency? If VSG length is a determinant of VSG activation, why is this different between silent VSGs in the expression sites and in the minichromosomes?
R3.4: As detailed above, our data do not support the view that 'VSG length is a determinant of VSG activation' (see R3.1), while the data presented in Fig 3D support the view that VSG length has a (statistically significant) impact on the competition among variants (see R3.3). The text beginning 'What is less clear…' has now been removed, since we acknowledge that we observed only 'a weak correlation between length and abundance changes for MC-*VSGs* (R^2=0.14; p=0.1)'. Other text from this paragraph has been incorporated elsewhere in the Discussion (see highlighted text in the third and fifth paragraphs).

3.5: A further complication, acknowledged by the authors in the results but ignored in the wider paper, is that relationship between VSG length and activation/growth efficiency (as detailed above and in Fig.3D) is only seen when comparing cells 9 and 5 days after Cas9 induction; Fig. 4E shows that no such length effect is seen when comparing days 9 and 13. This is not explained and, furthermore, there is not attempt to analyse the data across the full range of times that were sampled (up to days 17 and 25).
R3.5: Differences observed at earlier time-points, and not later, may be explained by 'the loss of clones that activate ES-*VSGs*', as stated in the 'Differential growth…' Results section. To address the latter point, we have now commented in the Fig 4E legend on R^2 and p-values for additional comparisons across the full range of times sampled. The values are, for day-17/13 (ES-*VSGs*: R^2=0.066, p=0.33; MC-*VSGs*: R^2=0.005, p=0.77), day-25/17 (ES-*VSGs*: R^2=0.03, p=0.52; MC-*VSGs*: R^2=0.007, p=0.73).

3.6: 2. The authors suggest that the effect of the length of an expressed VSG on growth can be explained by transcriptome differences in cells expressing long and short VSGs: e.g. (abstract) 'Differential growth of switched clones was also associated with wider transcriptome differences, affecting transcripts involved in nucleolar function, translation, and energy metabolism'. In the discussion, the authors present a more nuanced (and correct) argument: 'recently switched, and slower-growing trypanosomes expressing ES-derived VSGs displayed increased expression of genes involved in nucleolar function, protein translation and energy metabolism'. Here again, the significance of this finding is unclear. It is not explained why expression of different length VSGs would impose differential levels of changes to these aspects of the transcriptome, or why this is 'transient'. A possibility the authors appear not have not considered is that the transcriptome changes they detect at day 5 are due to the Cas9-induced break; either because some breaks are unrepaired, or lingering transcriptome effects of the cells responding to the break. The authors state 'we assessed growth rates at the earliest time-point possible'; when was this, and have they tested the timing of Cas9-induced break repair? Again, such information is needed to separate activation from growth.
R3.6: We accept that mechanistic connections between differential growth and transcriptome differences (or VSG length) remain to be elucidated. When considering why population level differences are transient, we do state in the 'Differential growth…' Results section, that growth differences 'may result in the loss of clones that activate ES-*VSGs*'. We did indeed consider the possibility that 'transcriptome changes … are due to the Cas9-induced break', but this would be inconsistent with the data shown in Fig 5E. To address this query further, however, we assessed our transcriptome data for changes in expression of 'DNA repair' genes (GO:0006281, n=86 genes), but found no significant differences in any of the six pairwise comparisons shown in Fig 5B (p=0.28 / 0.23) or 5E (p=0.67 / 0.58 / 0.33 / 0.59). This is consistent with our RNA-seq analysis being conducted after the VSG switching process was complete (see first paragraph in the 'RNA-seq…' Results section). In the case of unrepaired breaks, these would not be expected to yield a VSG switch (see Fig 1B). We had noted 'the earliest time-point possible' in the Materials and Methods section ('5 days after subcloning') and this is now also noted in the Results section.

Part III – Minor Issues: Editorial and Data Presentation Modifications

Reviewer #1:
1.4: VSG/RNA-seq mapping and analysis:
- Where did MC sequences come from? It seems like these are from the Cross "VSGnome" but the methods section only mentions the ES-VSGs and the 927 genome. This should be clarified.
R1.4: Apologies for this omission. We've now added further details in the Materials and Methods, 'RNA sequencing…' section.

1.5: - Similarly, were the only VSGs aligned to MC and ES VSGs? This potentially means that other (complete) VSGs in the genome could be coming up but aren't being evaluated by this analysis. If this is the case it should be noted somewhere.
R1.5: Further details have now been added to the 'RNA sequencing…' Materials and Methods section; 'Additional VSGs in preliminary analyses included subtelomeric array *VSGs* (*VSG-4* and *VSG-5*), but these *VSGs* failed to register sufficient reads to qualify as activated'.

1.6: - Why is the --very-sensitive-local option used for alignment instead of very sensitive in end-to-end mode? I know this has been shown to work well for BES mapping but I would worry that the soft clipping that (I think) is allowed in local mode could lead to incorrect mapping to some VSGs. Similarly, counting multi-mapping reads could lead to misleading results. Is there an advantage to counting this way? Since all comparisons are between multiple groups, and one VSG reference is used for all of the samples, it should be fine to count only uniquely

mapping reads. It's possible there are not very many multi-mapping reads occurring in the analysis presented; this could also be reported.
R1.6: With this option, the spliced-leader sequence should be soft-clipped rather than flagged as a chimaera that maps to different chromosomes. Also, there should be no multi-mapping since we map to truncated *VSGs* that lack shared sequences.

1.7: - I can't figure out how this conclusion is drawn from the RNA-seq data : "We also note here that despite frequent duplicative conversion of ES-VSGs, all ESs appear to encode unique VSGs, suggesting that cells with a duplicated VSG present in two ESs are often subsequently replaced or modified." I had to read this sentence quite a few times to understand what it was getting at, so the authors might also consider rephrasing.
R1.7: Apologies that this was unclear. We've now edited this text and added a citation towards the end of the 'RNA-seq revealed…' Results section.

1.8: VSG Length:
- Correlation of survival/establishment with length seems potentially overstated. Its worth discussing but I would be hesitant to present this as a major finding given the relatively weak association, particularly for the MC- VSGs. The finding that MC VSGs preferentially establish is very interesting on its own and much stronger.
R1.8: We agree and have adjusted the text in the Abstract accordingly.

1.9: - How does the distribution of lengths of the MC VSGs detected in these populations compare to the distribution across the whole MC VSG repertoire?
R1.9: The set of 20 MC *VSGs* analysed in our experiments were 485+/-25 aa in length (see Fig 2E), while an additional set of 25 MC *VSGs* were 477+/-30 aa in length. Although there is little difference here, we prefer not to include this information in our manuscript, since we are unable to assess the capacity of the latter set of genes to yield functional VSGs.

1.10: MC VSG Emergence:
- Do the authors think that the MC VSGs are early switchers that are establishing later in the population or later switch events that establish after they appear in the population? It sounds like the former explanation is favored, but one could imagine that with a different background of competing variants (VSG-2 is not an option any longer) that some variants could be switched to at later timepoints and then establish. Similarly, maybe these are derived from the large VSG-6 population, and their emergence is related to the prior expression of VSG-6. Either way, I think the very interesting observation that " the transition from ES-VSGs to MC-VSGs, previously thought to be driven by the adaptive immune response, in fact occurs in the absence of an adaptive immune response" would still hold. It just might be useful to be more explicit about how precisely these variants might emerge.
R1.10: A typical infection likely comprises a sufficient number of parasites to generate a large number of antigenic variants simultaneously (see the third paragraph of our Introduction). So we believe that many ES-*VSGs* and MC-*VSGs* are activated early in infection but come to prominence in the parasitaemia later (see the end of our Discussion).

1.11: Data Presentation:
- Figure 4 - There is a mistake in the figure- looks like a screenshot was taken while mousing over something?
R1.11: Apologies, this has been corrected.

1.12: - Figure 5b - What is being shown on the y axis? I assume RPKM for day 9/25, but it's not stated explicitly. Same for all of the plots of this type.
R1.12: Apologies, these are 'log2 RPKM'. Detail has now been added.

1.13: -There is no link to the github/zenodo sites with code
R1.13: Apologies. These are now provided in the 'Data and materials availability' section.

Reviewer #2:
2.5: Could the authors explain how activation frequency was measured? RPKM / # days?
R2.5: We've edited this text towards the end of the 'RNA-seq revealed…' Results section to clarify; 'read-count in our earliest RNA-seq sample (day 5) then revealed differential activation frequencies for each *VSG* and for each cohort of *VSGs'* (also see R3.2 above). We also cite (Supplementary data 1) at this point in the text.

2.6: Fig 3A (PCA based on VSG-expression). Could the authors speculate why parasite populations of day 25 are closer to day 5 (in component #2) than parasites on days 9, 13 and 17?
R2.6: The first component carries most of the variance (66%), while the second component carries 20% of the variance (now indicated in Fig 3A). In the second dimension, we observed that *VSG-11* and *-3* primarily drive the day-9, -13 and -17 populations apart from the day-5 populations, while *VSG-1954* and *-15* primarily drive the day-25 populations apart from the day-9, -13 and -17 populations.

2.7: The author summary is very clear!
R2.7: Thank-you.

Reviewer #3:
3.7: 3. A large part of the discussion is concerned with untested contributions of 70 bp repeat length to VSG switching: 'Although the length of 70-bp repeat tracts remains unknown at many sites, we found that the frequency of VSG activation was broadly consistent with the length of these tracts. Indeed, VSG-14 and VSG-15, the polycistronic ES-associated VSGs with the shortest adjacent tracts of 70-bp repeats [7], were activated at a lower frequency than any other polycistronic ES-associated VSG.' Have the authors determined the lengths of the repeats in these loci in their cells?
R3.7: We have not determined the lengths of the *VSG-14* and *VSG-15* associated repeats in our cells but feel that this is a valid discussion point relating to differential activation rates. We have now adjusted the text, however, to be clear that different length 70-bp repeat tracts we refer to were reported previously.

3.8: 4. Another focus of the discussion is mosaic VSG formation, but this seems rather a stretch since they have excluded all but full-length VSGs from their analysis. Can the authors provide any evidence that mosaic VSGs are present in the VSG expression sites after Cas9 switch induction?
R3.8: We do not have evidence that mosaic *VSGs* are present in the *VSG* expression sites after Cas9 switch induction, and indeed did not expect to observe mosaic *VSGs*, which are thought to require multiple recombination steps and to arise at low frequency. We feel that this is a valid discussion point relating to mosaic *VSG* formation, however. Although our experiments were not designed to assess or sample mosaic *VSGs*, our results suggest a potential solution to the long-standing conundrum of how and at what sites, mosaic *VSGs* are assembled.
